# Dielectric Constant and Conductivity of Blood Plasma: Possible Novel Biomarkers for Alzheimer's Disease

**DOI:** 10.1155/2020/5756382

**Published:** 2020-02-13

**Authors:** Ernest Dallé, Musa V. Mabandla, William M. U. Daniels

**Affiliations:** ^1^School of Laboratory Medicine and Medical Sciences, College of Health Sciences, University of KwaZulu-Natal, Durban 4000, South Africa; ^2^Faculty of Medicine and Biomedical Sciences, University of Yaoundé, Yaoundé, Cameroon; ^3^School of Physiology, University of the Witwatersrand, Johannesburg, South Africa

## Abstract

Alzheimer's disease is a complex debilitating neurodegenerative disease for which there is no cure. The lack of reliable biomarkers for Alzheimer's disease has made the evaluation of the efficacy of new treatments difficult and reliant on only clinical symptoms. In an aged population where cognitive function may be deteriorating for other reasons, the dependence on clinical symptoms is also unreliable. However, it is well established that infusion of *β*-amyloid into the dorsal hippocampus of rats leads to cognitive impairment in a rat model of Alzheimer's disease. Moreover, the blood plasma of *β*-amyloid-lesioned rats exhibits a distinct variation of the dielectric constant and conductivity when compared to that of normal rats in a time-dependent manner. These two electric parameters of blood plasma may therefore act as potential biomarkers for dementia due to Alzheimer's disease. This review is aimed at highlighting evidences that support blood plasma electrical properties, e.g., dielectric constant and conductivity as possible novel biomarkers for the early development and progression of dementia due to Alzheimer's disease.

## 1. Introduction

Alzheimer's disease (AD) is the most common form of dementia, currently affecting 75 million people worldwide and is projected to increase to 135 million individuals by 2050 [1–3]. The disease is mostly characterized by a progressive mental deterioration manifested by loss of memory, impaired cognitive ability and visual-spatial orientation, confusion, and disorientation [4–6]. These mental abnormalities greatly interfere with normal activities of daily living and can progress to a level where the patient is unable to perform basic life activities such as bathing, dressing, or even eating [7]. At an advanced stage of the disease, for example, patients cannot move, communicate, or recognize family members [7]. However, although patients may exhibit symptoms differently, common to all patients is forgetfulness of new information, e.g., dysfunction of short-term memory [8].

There are two forms of AD. The first is classified as early-onset Familial Alzheimer's disease (FAD) which is caused by autosomal dominant mutations in either amyloid precursor protein (APP) or presenilin 1 or 2 (PS1/PS2) gene [9–11]. It accounts for approximately 2% of all AD cases. FAD is known to be entirely inherited and extremely rare. The other form is Sporadic Alzheimer's disease (SAD) which may either be (a) early-onset, where symptoms appear before 60 years, or (b) late-onset which is more common and occurs after 60 years of age [8, 12]. SAD accounts for approximately 98% of all AD cases and despite the high prevalence, to date, its underlying etiology remains elusive.

Clinically, the diagnosis of cognitive decline and memory loss associated with AD is problematic as very often, these symptoms only appear at advanced stages of the disease when it is too late to reverse or meaningfully retard its progression [8, 13]. Moreover, accurate diagnosis requires experts with sophisticated equipment who are not always available and not affordable for all patients. The consequences of this being that many patients are often under or misdiagnosed.

Considering the difficulties surrounding AD clinical diagnosis, efforts involving the use of imaging techniques such as magnetic resonance imaging (MRI) and positron emission tomography (PET) are underway to improve the assessment of brain structure and function [1]. According to Mattsson et al. [14], these techniques may identify persons at risk for AD prior to the development of symptoms. However, translation of the benefits of these techniques to a large public has not yet materialized due to the high cost and/or the limited access to the equipment (PET, MRI) and mostly to the relative invasiveness of sample collection, e.g., the cerebrospinal fluid [3, 15]. In the meantime, the irreversibility of AD has prompted current treatment strategies to focus on drugs that can alleviate cognitive symptoms by improving basal forebrain cholinergic functioning. This approach is based on the hypothesis that AD results from a cholinergic deficit in brain regions (hippocampus, cortex) involved in memory [16, 17]. Therefore, in order to increase cholinergic transmission, inhibitors of the catabolic enzyme acetylcholinesterase are commonly prescribed for AD. Examples of these inhibitors are Tacrine, Physostigmine, Donepezil, Rivastigmine, and Galantamine [16, 17]. Other current drugs including Celastrol (which has antioxidant and anti-inflammatory activity), Memantine (an N-methyl-D-aspartate antagonist), or Selegiline (a monoamine oxidase inhibitor) have been used for AD treatment but still fail to stop the progression of the disease [8, 17–19].

Currently, there are ongoing experimental approaches that specifically focus on antiamyloid strategies because of the association of *β*-amyloid plaques and AD. These approaches include the development of a form of vaccine, inhibitors of the amyloidogenic enzymes *γ*-secretase and *β*-secretase, or peptides that reduce the toxic effects of *β*-amyloid plaques [20–24]. Thus far, the outcomes of these studies all agree with the fact that biomarkers for abnormal *β*-amyloid accumulation in the brain (hippocampus, cortex) may be a focal point to tackle AD associated with cognitive and memory dysfunction [17]. Physiologically, *β*-amyloid (A*β*) is produced following cleavage of the amyloid precursor protein (APP) which is a substrate for the aspartyl proteases *α*-secretase, *β*-secretase, and *γ*-secretase. The accumulation of the neurotoxic isoforms of A*β* protein (A*β*_42_) in the brain is the cause of plaques and neurofibrillary tangles that eventually causes the death of neurons in AD pathology [25–27]. Measurement of A*β*_42_ in the cerebrospinal fluid (CSF) has been found to be useful to clinicians to stage or monitor the development of AD. Moreover, examination of aminotruncated products of A*β*_42_ peptides compared to A*β*_42_ alone has improved the ability to differentiate a stable mild cognitive impairment (MCI) from that of those progressing to AD [28]. However, while a whole body of work exists that indicates a clear link between an abnormal cholinergic system, altered APP metabolism, and AD pathogenesis, it is likely that other mechanisms exist that contribute to the development of AD. This may explain why there are patients with significant and abnormal A*β* plaques and neurofibrillary tangle deposition in their brain, and yet, they do not exhibit any symptoms of AD [29]. Moreover, the literature also points out the genetic predisposition such as mutations within the APP gene, which have been proposed as causal factors for AD [30, 31]. However, without neglecting the considerable advances made by preclinical studies and early drug discovery in our understanding of AD pathogenesis, the repeated failures at phase III are gradually denting the confidence of both patients and researchers. The question that remains is to know if it is our understanding of the disease that is erroneous or the search in developing reliable drugs that is scarce. There is therefore an urgent need for widening not only the development of therapeutic approaches for AD but also the search for novel biomarkers that combine reliability, specificity, and affordability. The present review is aimed at highlighting evidences that support blood plasma electrical properties, e.g., dielectric constant and conductivity as possible novel biomarkers for dementia due to Alzheimer's disease.

## 2. Biomarkers

Biomarkers are molecules or substances that can help to state a normal or abnormal health condition. Biomarkers may, therefore, serve as indicators of normal biological processes, pathogenic processes, or pharmacologic responses to a therapeutic or health care intervention [32]. It is accepted that anything measured in a biological system *in vivo* or *in vitro* may function as a biomarker. Because of this possibility, several studies have investigated the usefulness of plasma proteins to predict the development of a disease [27, 33, 34]. An ideal biomarker is expected to be binary, i.e., absent in a healthy individual, only present in the disease state, and increasing with the severity of the disease [35]. To be used in the diagnosis and treatment of a disease, a good biomarker must be sensitive and specific. However, despite the promise of biomarkers as encapsulated within its theoretical definition, reality has shown that the identification of a reliable biomarker remains a difficult undertaking.

The complexity of biomarker discovery is clearly demonstrated by the variety of molecular and biochemical approaches which can be used to identify a biomarker, reliant on the biological sample available. For example, omics systems that include transcriptomics, proteomics, genomics, and metabolomics can be employed on samples such as cerebrospinal fluid, plasma, or autopsy tissue to detect novel biomarkers or a new pattern of biomarkers [36, 37]. However, the lack of reliable biomarkers for many complex brain diseases today suggests that these approaches are not as successful as previously expected. Concerning AD especially, an ideal early detection of different types of dementia requires simple, noninvasive, and nonexpensive diagnostic tests. Fortunately, compared to traditional methods used at present, the collection and measurement of the dielectric constant and conductivity of blood plasma are simple, less invasive, and nonexpensive. In addition, the dielectric constant and conductivity of blood plasma fulfils specific criteria such as (1) reflect the aging, the pathophysiological, or any pharmacological process in the brain; (2) highly sensitive and specific; (3) reproducible results over time changes; (4) clear cut-off values with at least twofold changes; and (5) easy collectible results and affordable tests. Since these criteria are the cornerstone of good biomarkers universally accepted by researchers, measuring the dielectric constant and the conductivity of blood plasma may be an ideal potential candidate in the search of novel biomarkers for AD [36]. In view of this reality, the present review chose to evaluate an alternative strategy in biomarker discovery that is based on the electrical properties of a biological fluid such as blood plasma.

## 3. Biomarkers for Alzheimer's Disease (AD)

**Figure 1:**
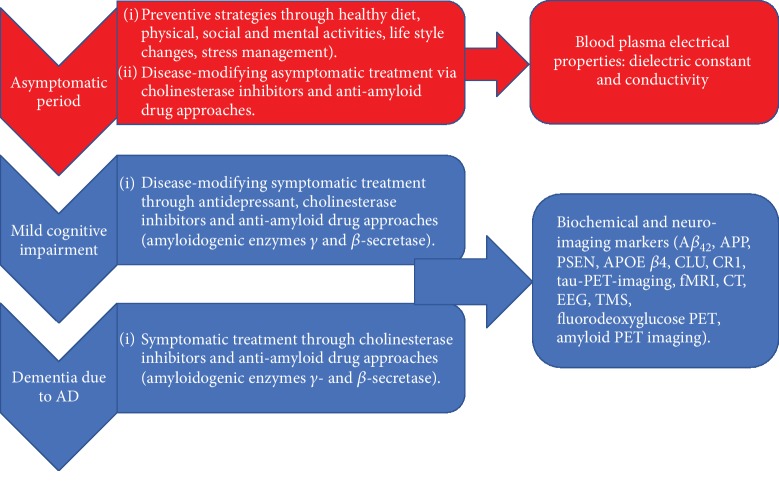
The various stages of AD and some approaches that can be adopted in identifying a reliable biomarker for AD. APP: amyloid precursor protein; PSEN: presenilin; APOE *β*4: apolipoprotein E *β*4; CLU: clusterin; CR 1: complement receptor 1; PET: positron emission tomography; fMRI: functional magnetic resonance imaging; CT: computerized tomography; EEG: electroencephalogram; TMS: transcranial magnetic stimulation.

Alzheimer's disease (AD), as is the case with many other neurodegenerative diseases, remains incurable but predictable [1, 38]. Consequently, the main approach to managing the disease is to diagnose it as early as possible and then apply the most effective therapies that will retard the progression (Figure 1).

However, the early detection is also problematic, hence the continued search for an ideal biomarker that may assist in identifying people at risk, give information about the evolution of the disease, or predict the response and toxicity to a given treatment. Some possible biomarkers for AD have been suggested. Amyloid precursor protein (APP), amyloid-beta (A*β*), presenilin (PSEN), apolipoprotein E *β*4 (APOE *β*4), clusterin (CLU), and complement receptor 1 (CR 1) are a few of the most important proteins present in the CSF that have been considered to serve as biomarkers [36, 37]. These proteins have all been linked to AD development and have etiological factor status. Other proteins such as ICAM-1 (intercellular adhesion molecule-1), VCAM-1 (vascular cell adhesion molecule-1), CT-proET-1 (C-terminal endothelin-1 precursor fragment), MR-proADM (midregional proadrenomedullin), or MR-proANP (midregional proatrial natriuretic peptide) have also been evaluated as candidates [5]. These proteins act on the microvascular system of the brain and may have an effect in blood vessel abnormalities observed in the central nervous system of patients with AD [5].

Therefore, in the face of difficult neuropsychological examination and mental status testing, tracking biomarkers through a simple blood test should be supportive to predict an early onset of the disease, especially in its asymptomatic stage which includes dementia. With the advantage of being less invasive, biochemical analyses of blood have the potential to help diagnose AD if regular blood test screening is recommended to people around sixty years old [39]. It is known that the brain of a patient with AD shows degeneration of the cholinergic neurons of the basal forebrain. Patients with AD also depict several distinct neuropathological dysfunctions including A*β* plaques and neurofibrillary tangles [16, 40]. However, these pathological features of AD precede the development of cognitive impairment (dementia) and therefore mark an advanced stage of the disease [41]. The situation is further complicated by the fact that not all patients who suffer from mild cognitive impairment (MCI) go on to develop AD. The benefit of novel biomarkers can therefore not be emphasized enough as they can provide accurate, specific, and reliable information concerning the disease stage. Thus, the discovery of predictive, reliable, biological markers for AD has become a major goal of many laboratories worldwide.

## 4. Dielectric Constant and Conductivity of Biological Samples

Amongst the various blood tests that have been used in studies exploring biomarkers in AD, the less widely used are the dielectric constant and the conductivity. The dielectric constant gives a measure of the polarizability of the material and therefore its ability to store charge [42]. The conductivity of a biological sample arises mainly from the mobility of the constituents (hydrated ions) present in the sample and therefore gives a measure of the ability of the sample to conduct a charge applied to it [43]. Dielectric constant and conductivity are therefore well-known measures of the physiological structure of a sample, which can accurately estimate the electromagnetic properties of that biological sample via the cavity perturbation technique [44–47]. For instance, blood plasma exposed to resonant frequencies adjusted for a maximum perturbation (2000 MHz to 4000 MHz of S-band in the microwave range) is able to reveal their exact constituents [48, 49]. In fact, the theory behind this is that, when blood plasma is introduced into a resonant cavity, the cavity field distribution and resonant frequency are expected to change depending on the biological constituents of the sample. According to the theory of cavity perturbation, the complex frequency shift is related according to [50]:

(1) $$\begin{array}{c} -\frac{\text{d}\Omega }{\Omega }\;\approx \;\frac{\left(\varepsilon_{r}\right)\;\int_{V_{s}}\;E\cdot \;E_{0}^{*}\;dV\;}{2\int_{V_{c}}{\left|{}_{E_{0}}\right|}^{2}\;dV}, \end{array}$$

(2) $$\begin{array}{c} But\frac{d\Omega }{\Omega }\;\approx \;\frac{d\omega }{\omega }+\;\frac{j}{2}\;\left[\frac{1}{Q_{s}}\;-\;\frac{1}{Q_{0}}\right]. \end{array}$$

Equating (1) and (2) and separating real and imaginary parts results in

(3) $$\begin{array}{c} {\varepsilon_{r}}^{/}-1\;=\;\frac{f_{o}-f_{s}}{2\;f_{s}}\left(\frac{V_{c}}{V_{s}}\right),\\ {\varepsilon_{r}}^{//}\;=\;\frac{V_{c}}{4\;V_{s}}\left(\frac{Q_{o}-Q_{s}}{Q_{o}Q_{s}}\right), \end{array}$$

where $\overset{¯}{\varepsilon_{r}}\;=\varepsilon_{r}^{/}\;-\;j\;\varepsilon_{r}^{//}$, $\overset{¯}{\varepsilon_{r}}$ is the relative complex permittivity of the sample, *ε*_*r*_^/^ is the real part of the relative complex permittivity, which is known as the dielectric constant. *ε*_*r*_^//^ is the imaginary part of the relative complex permittivity associated with the dielectric loss of the material. *V*_s_ and *V*_c_ are corresponding volumes of the sample and the cavity resonator. The conductivity can be related to the imaginary part of the complex dielectric constant as follows:

(4) $$\begin{array}{c} \sigma_{e}\;=\;{\omega \varepsilon }^{//}\;=\;2\pi \;f\;\varepsilon_{0}\varepsilon_{r}^{//}. \end{array}$$

Since the electromagnetic properties of biological samples including blood plasma are dependent on their constituents, a clear difference between a normal and an abnormal blood sample is therefore expected.

The measurement of the electrical properties of biological samples can be an ideal novel way to assess unique properties of a substance under investigation. While this method is highly appreciated in engineering and biophysics fields, its application in medical sciences in general remains underexploited [43]. Only a few studies have focused on the conductivity property and dielectric constant of biological samples but have reported interesting and promising results [48–53]. These studies have demonstrated that blood plasma conductivity and dielectric constant of HIV-/AIDS-infected patients, A*β*_42_ chemically induced sample, or even infected mucus of the H1N1 virus that causes human influenza exhibits different behaviors than that of normal blood [48, 50–53]. The application of specific microwave frequencies to measure the dielectric constant and conductivity has thus far allowed researchers to differentiate normal than abnormal human colostrum [50], human semen [51], and blood of patients infected with the HIV/AIDS and H1N1 viruses [52, 53]. Moreover, the dielectric properties of blood plasma have also been used to indicate biomass, electrokinetic separation, and characterization of single cells [42]. The recent study by Lonappan et al. [49] found a substantial difference in the dielectric properties of A*β*_42_ chemically induced blood samples when compared to those of normal samples. In this study, the authors were able to demonstrate that determining the dielectric constant and the conductivity of blood plasma may be useful biomarkers of abnormalities in learning and memory associated with AD [49]. Despite these promising findings, data concerning the dielectric constant and conductivity of most tissues are still either very limited or nonexistent; hence, they need to be generated [54]. This scarcity of information may be tackled if the widely available and sophisticated microwave equipment commonly housed within engineering departments is increasingly used by medical scientists. The limited preclinical and clinical studies that can validate data found so far need to be amplified in order to recognize the dielectric constant and conductivity of blood plasma as reliable biological markers of AD abnormality.

As blood plasma is a heterogeneous medium with proteins as one of its main constituents, its electrical properties (behavior) may therefore closely reflect its physiological composition [48, 55]. It is possible to prevent AD even though it is still impossible to cure this disease [38]. Current palliative treatments available for AD may be more useful in the case of an early diagnosis via the measurement of electrical properties of blood plasma. The present paper therefore emphasizes that behavioral changes (early onset) in patients with AD may be reflected in the electrical properties of their blood plasma. Consequently, determining the electrical properties of blood plasma, for instance, the dielectric constant and conductivity of blood plasma, may be useful as ideal novel biomarkers for complex brain disease such as AD.

## 5. Conclusion

It is evident that A*β*_42_ induces abnormalities in learning and memory and can reflect some aspects of AD [49]. There is also evidence showing that changes in the dielectric constant and conductivity of blood plasma of patients at risk of AD can predict AD. Electrical properties of blood plasma and, for instance, their dielectric constant and conductivity are possible novel biomarkers which can help predict AD associated with learning and memory deficits as they suggest some degree of difference in blood composition.

## Abbreviations

A*β*: *β*-Amyloid
AD: Alzheimer's disease
AIDS: Acquired immune deficiency syndrome
APOE *β*4: Apolipoprotein E *β*4
APP: Amyloid precursor protein
CLU: Clusterin
CR1: Complement receptor 1
CSF: Cerebrospinal fluid
CT: Computerized tomography
CT-proET-1: C-terminal endothelin-1 precursor fragment
EEG: Electroencephalogram
FAD: Familial Alzheimer's disease
fMRI: Functional magnetic resonance imaging
H1N1: Hemagglutinin-1 neuraminidase-1
HIV: Human immunodeficiency virus
ICAM-1: Intercellular adhesion molecule-1
MCI: Mild cognitive impairment
MRI: Magnetic resonance imaging
MR-proANP: Midregional proatrial natriuretic peptide
MR-proADM: Midregional proadrenomedullin
PET: Positron emission tomography
PS1: Presenilin 1
PS2: Presenilin 2
PSEN: Presenilin
SAD: Sporadic Alzheimer's disease
TMS: Transcranial magnetic stimulation
VCAM-1: Vascular cell adhesion molecule-1.
